# Optimizing time-limited non-pharmaceutical interventions for COVID-19 outbreak control

**DOI:** 10.1098/rstb.2020.0282

**Published:** 2021-07-19

**Authors:** Alex L. K. Morgan, Mark E. J. Woolhouse, Graham F. Medley, Bram A. D. van Bunnik

**Affiliations:** ^1^ Centre for Immunity, Infection and Evolution and School of Biological Sciences, University of Edinburgh, Edinburgh, UK; ^2^ Usher Institute, University of Edinburgh, Edinburgh, UK; ^3^ Centre for Mathematical Modelling of Infectious Diseases and Department of Global Health and Development, London School of Hygiene and Tropical Medicine, London, UK

**Keywords:** COVID-19, modelling, optimization, epidemiology/non-pharmaceutical interventions

## Abstract

Retrospective analyses of the non-pharmaceutical interventions (NPIs) used to combat the ongoing COVID-19 outbreak have highlighted the potential of optimizing interventions. These optimal interventions allow policymakers to manage NPIs to minimize the epidemiological and human health impacts of both COVID-19 and the intervention itself. Here, we use a susceptible–infectious–recovered (SIR) mathematical model to explore the feasibility of optimizing the duration, magnitude and trigger point of five different NPI scenarios to minimize the peak prevalence or the attack rate of a simulated UK COVID-19 outbreak. An optimal parameter space to minimize the peak prevalence or the attack rate was identified for each intervention scenario, with each scenario differing with regard to how reductions to transmission were modelled. However, we show that these optimal interventions are fragile, sensitive to epidemiological uncertainty and prone to implementation error. We highlight the use of robust, but suboptimal interventions as an alternative, with these interventions capable of mitigating the peak prevalence or the attack rate over a broader, more achievable parameter space, but being less efficacious than theoretically optimal interventions. This work provides an illustrative example of the concept of intervention optimization across a range of different NPI strategies.

This article is part of the theme issue ‘Modelling that shaped the early COVID-19 pandemic response in the UK’.

## 1. Introduction

The ongoing COVID-19 pandemic has highlighted the vital role of non-pharmaceutical interventions (NPIs) in mitigating the spread of SARS-CoV-2. These interventions aim to break chains in transmission through population-and individual-level behavioural changes, which can consequently reduce opportunities for transmission [1]. NPIs encompass a large range of potential outbreak control strategies, ranging from simple advice to encourage hand-washing to country-wide, severe ‘lockdown’ measures such as stay-at-home orders, mobility restrictions and closure of non-essential businesses [2].

While an effective tool to drive down disease prevalence, severe NPIs are considered unsustainable and time-limited, with economic, physical and mental health repercussions during and following the cessation of these interventions [3–5]. This has driven calls to retrospectively understand the epidemiological and human health impacts of introducing severe NPIs under a different set of circumstances [6–8]. This includes insight into how differences in the timing, duration and strength of these interventions could have potentially altered COVID-19-associated mortality and morbidity compared with the actual course of action.

Intervention optimization has been proposed as a method to allow policymakers to fine-tune the characteristics of an intervention to minimize epidemiologically relevant outcome measures. Optimization has been explored for a range of potential COVID-19 NPI strategies, including single time-limited reductions to transmission [9,10], intermittent pulsing of NPIs [11,12] and gradual ramping-down of intervention measures following an initial reduction to transmission [12–14]. This has been explored in the context of minimizing the peak incidence or prevalence, analogous to ‘flattening the curve’ of an outbreak.

Despite theoretically optimal interventions being identified in a number of optimization analyses for COVID-19, the ability for policymakers to achieve these results in practice has been questioned [10]. This stems from the narrow windows for optimal implementation, with minor deviations from the optimal intervention timing, duration or magnitude often greatly reducing the efficacy of the NPI and therefore having severe human health consequences [10,13]. This sensitivity to implementation error is likely to be amplified in emerging outbreak situations such as the COVID-19 pandemic, with imperfect epidemiological knowledge of uncharacterized, novel pathogens preventing policymakers from fine-tuning NPIs to a narrow optimal parameter space. An alternative strategy is to use generalized intervention strategies that are, as an example, longer or earlier than the theoretically optimal parameter space [15]. Such interventions can be denoted ‘robust’ interventions. The rationale behind these pragmatic interventions would be to identify a broad and achievable parameter space that may be suboptimal, but potentially more robust to implementation error, while still capable of mitigating the detrimental epidemiological impacts of COVID-19 [10]. Owing to the robust and general nature of these interventions, they also offer more practical and flexible guidance to policymakers than specific optimal intervention timings or durations.

This study aims to provide a mathematical modelling framework to explore the concept of optimal and robust interventions across a range of different NPI scenarios. We explore and compare the existence, patterns and optimal parameter spaces for each intervention to minimize the peak prevalence or the attack rate of a simulated outbreak. This is explored for three main parameters: (i) intervention duration, (ii) intervention strength, and (iii) the intervention trigger point. The results from this study are not intended to highlight an absolute best course of action. Rather, this analysis provides an illustrative example to describe how optimal and robust outbreak control can be achieved under different circumstances and intervention strategies.

## 2. Methods

### (a) Susceptible–infectious–recovered model structure

A deterministic susceptible–infectious–recovered (SIR) model [16] was used to explore the impact of time-limited NPIs on a simulated UK-based COVID-19 outbreak. *S*, *I* and *R* compartments denote the fraction of susceptible, infectious and recovered individuals, respectively, within the population (equation 2.1)

2.1 $$\begin{array}{rl} \frac{dS}{dt}=\; & -\beta (t)SI,\\ \frac{dI}{dt}=\; & \beta (t)SI-\gamma I\;\\ \mathrm{and}\;\;\;\frac{dR}{dt}=\; & \gamma I\;. \end{array}\}$$

Susceptible individuals (*S*) were infected at the time-varying rate *β*(*t*), representing the daily *per capita* rate of transmission in a randomly mixing population. Infectious individuals (*I*) were assumed to recover at rate *γ*, representing the daily *per capita* rate of recovery. This rate was taken as the inverse of the average duration of infectiousness. A baseline pre-NPI basic reproduction number (*R*_0_) of 2.8 and doubling time (*T*_d_) of 3 days were assumed [17–21]. The generation time was calculated as a function of these two quantities [22], with a baseline generation time of 7.79 days and a resulting *γ* of 0.128 day^−1^ (equation 2.2)

2.2 $$\mathrm{Generation}\;\;\mathrm{time}\;=T_{d}\frac{\left(R_{0}-1\right)}{\ln2}.$$

### (b) Defining the time-varying *β*(*t*)

By setting *β* = *R*_0_*γ*, we defined the baseline *per capita* transmission rate in the absence of NPIs, *β* = 0.359 day^−1^. To capture the impact of smaller scale NPIs, *β* was multiplied by a scaling factor of 0.7, *β*_scale_ = 0.252 day^−1^, with this 30% reduction being in line with estimates of the impact of NPIs, such as school-closures, introduction of social distancing and isolation upon COVID-19 symptoms and excluding severe NPIs, such as stay-at-home orders [21,23,24]. We assumed that these measures were in place at the initiation of the model simulation. Using the UK as a representative example, these measures were introduced between 12 and 21 March 2020, with severe ‘lockdown’ measures initiated on 25 March 2020 [24]. However, it was not the intention of this study to model the exact timing of the UK outbreak response, rather to use the epidemiological characteristics of the UK outbreak as motivation for this study.

*β*(*t*) was defined as the product of *β*_scale_ and a time-varying scaling factor *c*(*t*), which reduced *β*_scale_ over the course of the simulation to model the impact of severe NPI measures, with 0 ≤ *c*(*t*) ≤ 1 (equation (2.3)). Reductions associated with this scaling factor were introduced at the intervention trigger point, *t*_p_, and with *d_t_* describing the duration of the intervention

2.3 $$\beta (t)=\{c(t)\begin{array}{cc} \beta_{\mathrm{scale}}, & \;t<t_{p}\\ \beta_{\mathrm{scale}}, & \;t_{p}\leq t\leq t_{p}+d_{t}\\ \beta_{\mathrm{scale}}, & \;t>t_{p}+d_{t}. \end{array}$$

The shape of the *c*(*t*) factor varied with the different intervention scenarios explored, with parameter *c*_min_ describing the minimum value of *c*(*t*) during the intervention. This can be considered a proxy measure of the magnitude of the intervention. For baseline reductions to *β*(*t*), we defined *c*_min_ = 0.4, resulting in *β*(*t*) = 0.101 when the NPI measures are at their greatest magnitude*.* Baseline *c*_min_ was chosen to roughly achieve an effective reproduction number (*R*_e_) of 0.7 ≤ *R*_e_(*t*) ≤ 1 during the intervention [21,23,24], with *R*_e_(*t*) defined as *R*_0_*S*(*t*). All interventions were initiated at baseline *t*_p_ = 52 days, equivalent to an attack rate at the initiation of the severe NPI measures of *I_c_*(52) = 0.02, in line with model-based UK COVID-19 estimates [24]. The model was seeded with an initial infectious fraction *I*(0) = 0.00001.

### (c) Single period of severe non-pharmaceutical interventions

A time-limited period of severe NPI measures was the primary intervention explored in this model, with optimization occurring in relation to this intervention. We explored five different intervention scenarios, with each scenario differing with regard to the shape of *c*(*t*) and the subsequent *β*(*t*) reductions over the duration of the intervention (*d_t_*) (table 1). The total duration of the simulation, *t*_max_, was set at 400 and 1000 days for all other sensitivity analyses. We provide the rationale and real-world parallels for each scenario in the electronic supplementary material, p. 1.

**Table 1.** Description of the five intervention scenarios.

scenario	description of *c*(*t*)	definition of *c*(*t*)
1	immediate and constant reduction to *c*_min_	$c(t)=c_{\min}$
2	immediate reduction to *c*_min_ followed by a linear increase back to *c*(*t*) = 1	$c(t)=c_{\min}+\frac{1-c_{\min}}{d_{t}}(t-t_{p})$
3	linear decrease to *c*_min_ followed by an immediate return to *c*(*t*) = 1	$c(t)=1-\frac{1-c_{\min}}{d_{t}}(t-t_{p})$
4	linear decrease to *c*_min_ at *d_t_*/2, followed by a linear increase back to *c*(*t*) = 1	$c(t)=\{\begin{array}{c} \;1-\frac{1-c_{\min}}{d_{t}/2}(t-t_{p}),\;\;t_{p}\leq t<t_{p}+\frac{d_{t}}{2}\\ c_{\min}+\frac{1-c_{\min}}{d_{t}/2}(t-t_{p}),\;t_{p}+\frac{d_{t}}{2}\leq t\leq t_{p}+d_{t} \end{array}$
5	a ‘pulsing’ intervention with immediate reductions to *c*_min_ between intervention intervals 0–21, 35–49 and 63–77 days (for an example total intervention duration, *d_t_* = 84 days)	$c(t)=\{\begin{array}{cc} c_{\min}, & t_{p}\leq t<t_{p}+\frac{1}{6}d_{t}\\ 1, & t_{p}+\frac{1}{6}d_{t}\leq t<t_{p}+\frac{2}{6}d_{t}\\ c_{\min}, & t_{p}+\frac{2}{6}d_{t}\leq t<t_{p}+\frac{3}{6}d_{t}\\ 1 & t_{p}+\frac{3}{6}d_{t}\leq t<t_{p}+\frac{4}{6}d_{t}\\ c_{\min}, & t_{p}+\frac{4}{6}d_{t}\leq t<t_{p}+\frac{5}{6}d_{t}\\ 1 & t\geq t_{p}+\frac{5}{6}d_{t} \end{array}$

For a given intervention duration, *d_t_*, the magnitude of *c*(*t*) scaling reductions over the intervention duration was half for scenarios 2, 3, 4 and 5 relative to scenario 1. To maintain comparable *β*(*t*) reductions over the intervention period, *d_t_* was doubled for scenarios 2, 3, 4 and 5 relative to scenario 1 for baseline analyses. This corresponds to *d_t_* = 84 days for scenario 1 (12 weeks) and *d_t_* = 168 days (24 weeks) for all other scenarios. However, we note this was not possible in sensitivity analyses where *d_t_* was an explored parameter. Instead, the *d_t_* range for scenario 1 was transformed into a relative axis to enable comparisons across scenarios. This was achieved by halving the explored *d_t_* range for scenario 1 relative to all other scenarios, and then doubling the absolute values in this limited *d_t_* range into a relative scale. As an illustrative example, an absolute value of *d_t_* = 125 is equal to a relative value of *d_t_* = 250 for scenario 1. Baseline parameter values for the modelling of single NPIs can be found in electronic supplementary material, table S1.

An alternative approach was considered by keeping *d_t_* constant and doubling *c*_min_ in scenarios 2, 3, 4 and 5 relative to scenario 1 (electronic supplementary material, figures S1 and S2). However, in practice, it is likely more plausible to alter *d_t_* than it is to alter *c*_min_ in a public health context. This can be attributed to difficulty faced by policymakers to introduce an intervention with a specific magnitude or strength [25].

### (d) Multiple non-pharmaceutical interventions

To explore the transmission dynamics resulting from multiple, time-limited periods of severe NPIs, two interventions were modelled for each scenario sequentially during the simulation. We defined the minimum value of the *c*(*t*) scaling factor, trigger point and duration of the intervention as *c*_min1_ and *c*_min2_, *t*_p__1_ and *t*_p__2_, and *d_t_*_1_ and *d_t_*_2*,*_ respectively, for interventions 1 and 2. We note that *t_*p*2_* is defined relative to the end of intervention 1, with the start of intervention 2 defined as *t* = *t*_p__1_ + *d_t_*_1_ + *t*_p__2_.

Baseline parameter values for the multi-intervention scenario were set at *d_t_*_1_ = *d_t_*_2_ = 42 days (6 weeks) for scenario 1 and *d_t_*_1_ = *d_t_*_2_ = 84 days (12 weeks) for scenarios 2, 3, 4 and 5. This was halved relative to the single-intervention scenarios to allow the two interventions to occur within the timeframe of the simulated outbreak and to prevent unfeasibly long overall intervention durations. We note that comparisons between the single-and multi-intervention scenarios should be limited to the general qualitative pattern of the optimal parameter space, rather than being direct quantitative comparisons. The minimum value of the scaling factor *c*(*t*) was kept constant at *c*_min1_ = *c*_min2_ = 0.4 at baseline. Baseline parameter values for the modelling of multiple NPIs can be found in electronic supplementary material, table S2.

### (e) Outcome measures of interest

The primary objective of all analyses in this study was to identify the optimal parameter space for the intervention trigger point (*t*_p_), duration (*d_t_*) and magnitude (c_min_) to minimize two outcome measures:

1. peak *I*(*t*) prevalence: $I_{\max}$_,_
2. attack rate: $I_{c}(t_{\max})\;=\underset{t\to t_{\max}}{\lim}I_{c}(t)$.

We defined *I*_max_ as the global maximum of the function describing the trajectory of the fraction infectious during the simulated epidemic, with subsequent references to ‘epidemic peaks’ describing the local maxima where *I*(*t*) > 0 and *I'*(*t*) = 0. The attack rate, *I_c_*(*t*_max_), was defined as the total proportion of cases that develop over the model simulation duration. The optimal parameter space was defined as the combination of parameter values that resulted in the lowest possible value of *I*_max_ or *I_c_*(*t*_max_).

### (f) Software used

All simulations were carried out using R [26] and RStudio [27]. R package ‘deSolve’ [28] was used for all model simulations. All other R packages used for plotting can be found in the electronic supplementary material.

## 3. Results

**Figure 1.**
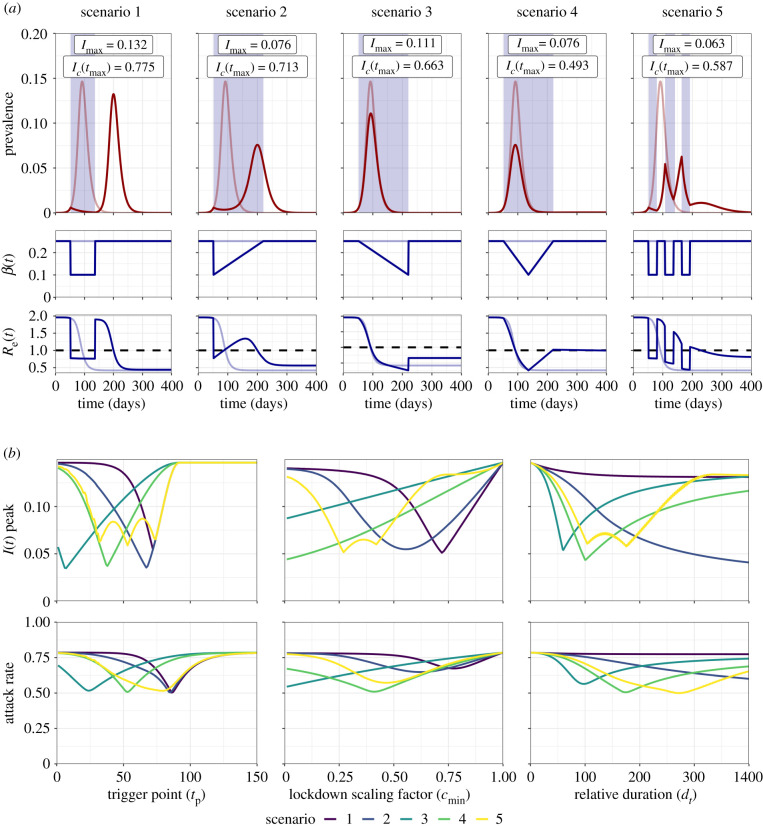
(*a*) Trajectory plots for the epidemic curve, *β*(*t*) reductions and *R*_e_(*t*) for the five intervention scenarios. (*b*) Sensitivity analysis for intervention trigger point (*t*_p_), magnitude (*c*_min_) and duration (*d_t_*) to minimize the peak prevalence, *I*_max_, and attack rate, *I_c_*(*t*_max_). For (*a*), pale red and blue lines depict unmitigated epidemic curve dynamics, blue shading indicates the intervention period and the dotted line depicts the *R*_e_(*t*) = 1.0 threshold for sustained epidemic growth. *I*_max_ and *I_c_*(*t*_max_) values are annotated for each scenario. Note that for (*b*), the *d_t_* axis for scenario 1 was transformed into a relative axis to allow comparison across scenarios, with the relative axis of 0 ≤ *d_t_* ≤ 400 being equal to an absolute *d_t_* range of 0 ≤ *d_t_* ≤ 200.

The impact of the five intervention scenarios on the trajectory of a simulated COVID-19 outbreak was explored (figure 1*a*). Scenario 4 was identified as the most effective scenario at mitigating peak prevalence or the attack rate under baseline parameters (*I*_max_ = 0.076, *I_c_*(*t*_max_) = 0.493) relative to an unmitigated outbreak (*I*_max_ = 0.146, *I_c_*(*t*_max_) = 0.786). Scenarios 1 and 2 resulted in the suppression of the initial outbreak and delay in the epidemic peak following the initiation of intervention measures. By contrast, a single mitigated epidemic peak was observed for scenarios 3 and 4, with the steady ramping up of *β*(*t*) reductions and the protective effects of population immunity resulting in a more gradual, sustained reduction to *R*_e_(*t*), preventing peak resurgence. The pulsed nature of scenario 5 allowed brief opportunities for the build-up of population immunity (*R*_e_(*t*) > 1) and subsequent epidemic control (*R*_e_(*t*) < 1).

Sensitivity analyses were conducted to observe the sensitivity of the peak prevalence, *I*_max_, and the attack rate, *I_c_*(*t*_max_), to the intervention trigger point (*t*_p_), magnitude (*c*_min_) and duration (*d_t_*) (figure 1*b*). As an initial exploration into model dynamics, each parameter was investigated sequentially with all other model parameters held constant at baseline values. Optimal parameter values for all scenarios can be found summarized in electronic supplementary material, table S3.

A specific optimal trigger point was observed for all scenarios to minimize both *I*_max_ and *I_c_*(*t*_max_), with these optimal values found within an early-intermediate trigger point parameter space (7 ≤ *t*_p_ ≤ 74). While an optimum was identified for scenario 5 to minimize *I*_max_ (*t*_p_ = 53), two other trigger points resulted in similar reductions to *I*_max_ (*t*_p_ = 32 and 74). Scenarios 1, 3 and 4 were found to be highly sensitive to deviations from the optimal *t*_p_ value, with steep increases in *I*_max_ and *I_c_*(*t*_max_) either side of the optimum. We note that if a suboptimal trigger point was chosen in scenario 2, it would be more beneficial to intervene earlier-than-optimal, with a gentler increase in *I*_max_ as *t*_p_ is reduced from the optimal value, compared with later-than-optimal *t*_p_ values.

Stronger interventions resulted in optimal reductions to *I*_max_ and *I_c_*(*t*_max_) for scenarios 3 and 4 (*c*_min_ → 0). By contrast, intermediate-strength interventions were found to be optimal in scenarios 1, 2 and 5 (*c*_min_ = 0.72/0.77, 0.56/0.62, 0.27/0.47) for *I*_max_/*I_c_*(*t*_max_), respectively. We note that if suboptimal intervention magnitudes were chosen for scenarios 1, 2 and 5, it was more beneficial to intervene too strongly than insufficiently. This was observed with decreases in *c*_min_ from the optimal value resulting in gentler increases in *I*_max_ and *I_c_*(*t*_max_) compared with greater-than-optimal *c*_min_ values.

Longer intervention durations were found to be optimal to reduce *I*_max_ and *I_c_*(*t*_max_) for scenario 2 (*d_t_* → 400). Interestingly, increasing the intervention duration was found to have minimal impact on either outcome measure in scenario 1, with the cessation of the intervention resulting in an identically sized epidemic peak regardless of the intervention duration. By contrast, intermediate-length interventions were found to be optimal for scenarios 3 and 4 (*d_t_* = 60/97, 100/174) for *I*_max_/*I_c_*(*t*_max_), respectively, with scenario 5 displaying two relatively similar optimal points to minimize *I*_max_ (*d_t_* = 104/175). We note that if a suboptimal intervention duration was introduced for these scenarios, it was better to intervene for too long, with increases in *I*_max_ and *I_c_*(*t*_max_) being less severe in an intervention that was longer-than-optimal, compared with an intervention that was shorter-than-optimal.

**Figure 2.**
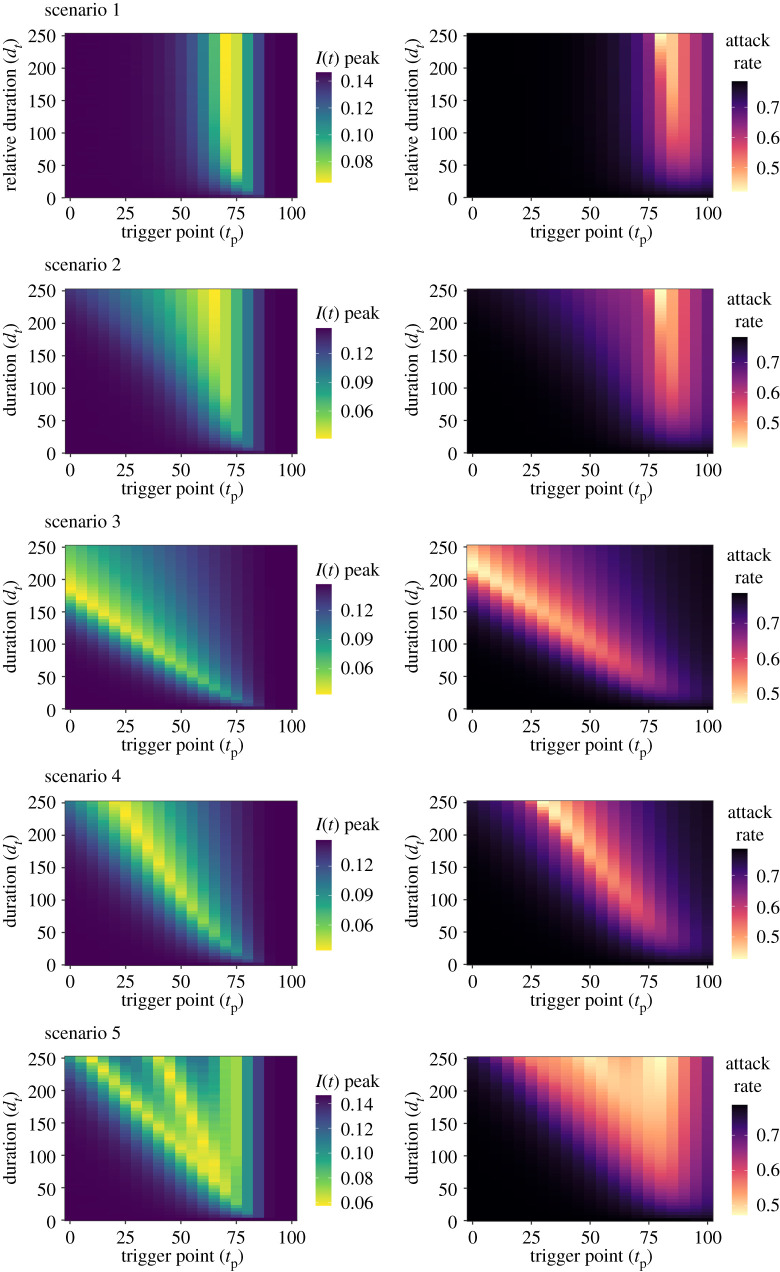
Sensitivity analysis for the peak prevalence, *I*_max_, and attack rate, *I_c_*(*t*_max_), for intervention trigger point, *t*_p_, and duration, *d_t_*. Note that the scenario 1 *d_t_* axis was transformed into a relative axis to allow comparison across scenarios, with the relative axis of 0 ≤ *d_t_* ≤ 250 being equal to an absolute *d_t_* range of 0 ≤ *d_t_* ≤ 125.

To explore the interplay between multiple model parameters, a sensitivity analysis was next conducted to identify the optimal parameter space to minimize *I*_max_ and *I_c_*(*t*_max_) for a multi-dimensional parameter space: (i) intervention trigger point (*t*_p_) and (ii) intervention duration (*d_t_*) (figure 2). The optimal parameter space for all scenarios can be found summarized in electronic supplementary material, table S3.

A longer intervention duration (*d_t_* → 250) and intermediate trigger point (*t*_p_ = 70/66 and *t*_p_ = 80) was optimal for scenarios 1 and 2, respectively, to minimize *I*_max_ and *I_c_*(*t*_max_). A contrasting pattern was observed in scenarios 3 and 4, with shorter intervention durations found to maintain a near-optimal parameter space with a later intervention trigger. We note the existence of suboptimal trigger point ‘gaps’ in scenario 5, with increases and decreases in *I*_max_ as the trigger point was varied. This resulted from the fixed periods between pulsed interventions, with these ‘gaps’ increasing as the duration of the overall intervention increased. These ‘gaps’ were found to be less pronounced for *I_c_*(*t*_max_) relative to *I*_max_. Increasing the duration of the intervention had compensatory effects for scenarios 2, 3, 4 and 5, with both *I*_max_ and *I_c_*(*t*_max_) becoming less sensitive to deviations from the optimal intervention trigger point as the duration of the intervention was increased. This suggests that increasing the duration can make the intervention more robust to optimal trigger point implementation error.

The sensitivity analysis was repeated with *c*_min_ = 0.25/0.5/0.75 to assess the sensitivity of the *d_t_*/*t*_p_ relationship to alterations to the magnitude of the intervention (electronic supplementary material, figures S3 and S4). Low-to-intermediate *c*_min_ values of 0.25 (scenarios 1, 2 and 3) and 0.5 (scenarios 3 and 4) were found to be optimal to minimize *I*_max_, with the lowest explored value of *c*_min_ being optimal to minimize *I_c_*(*t*_max_) for all scenarios.

Two sequentially implemented interventions for each of the five scenarios were next modelled to explore the introduction of NPIs at a later date to tackle epidemic resurgence. Descriptive trajectory plots for each of the five multi-intervention scenarios can be found in electronic supplementary material, figure S5. We note a higher value of *I*_max_ for scenarios 2 and 5 and the existence of an increased number of epidemic peaks for scenarios 1 and 4 relative to the single-intervention scenarios. This can be attributable to the shorter intervention duration used for the multi-intervention scenarios.

**Figure 3.**
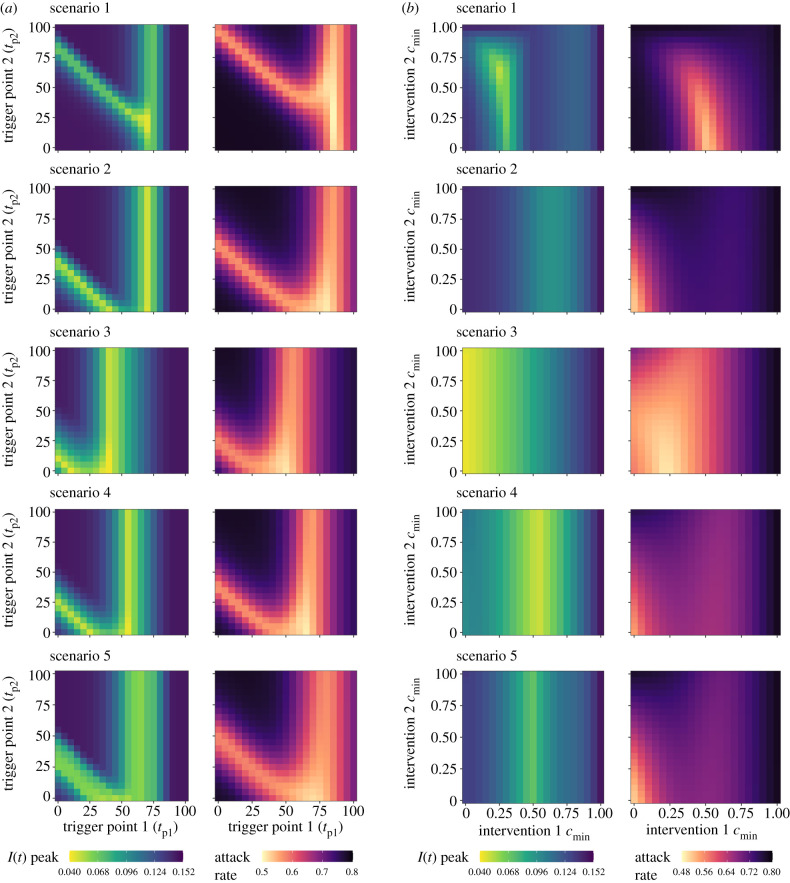
(*a*) Sensitivity analysis for the peak prevalence, *I*_max_, and attack rate, *I_c_*(*t*_max_), for intervention 1 trigger point, *t*_p__1_, and intervention 2 trigger point, *t*_p__2_. (*b*) Sensitivity analysis for the minimum value of scaling factor *c*(*t*) for intervention 1, *c*_min1_, and intervention 2, *c*_min2_.

A sensitivity analysis was conducted for the multi-intervention model to explore the optimal parameter space to minimize *I*_max_ and *I_c_*(*t*_max_) for two sets of parameters: (i) interventions 1 and 2 trigger points, *t*_p__1_ and *t*_p__2_*,* and (ii) interventions 1 and 2 magnitudes, *c*_min1_ and *c*_min2_ (figure 3). The optimal parameter space for all scenarios can be found summarized in electronic supplementary material, table S3.

A large range of trigger points for intervention 2 (1 ≤ *t*_p__2_ ≤100) were found to result in near-optimal reductions to *I*_max_ and *I_c_*(*t*_max_), on the condition that the optimal trigger point for intervention 1 was achieved (50 ≤ *t*_p__1_ ≤ 65) (figure 3*a*). This was found to differ if an earlier-than-optimal intervention 1 trigger point was chosen, with only a narrow selection of optimal intervention 2 trigger points able to compensate for a suboptimal *t*_p__1_ value. The choice of a later-than-optimal intervention 1 trigger was found to negate the ability for an intervention 2 trigger to prevent increases in *I*_max_ and *I_c_*(*t*_max_), suggesting that it is better to introduce the initial intervention earlier, rather than later, if the optimal intervention 1 trigger point is unknown. Extending the duration of intervention 1 and 2 did little to alter the optimal trigger points for all scenarios (electronic supplementary material, figures S6–S10).

A large range of intervention 2 magnitudes (0 ≤ *c*_min2_ ≤ 1) were found to provide near-optimal reductions to *I*_max_, on the condition that the magnitude of intervention 1 was sufficiently optimized for scenarios 2, 4 and 5 (figure 3*b*). This suggests that for these scenarios, it is critical to focus on optimizing the initial intervention to minimize *I*_max_. A different optimal parameter space was identified to minimize *I_c_*(*t*_max_) for these three scenarios, with strong reductions to both interventions 1 and 2 being favoured (*c*_min1_/*c*_min2_ → 0). Scenario 3 displayed subtly different dynamics, with intervention 1 ideally being as strong as possible to minimize *I*_max_ (*c*_min1_ → 0) and an intermediate magnitude to minimize *I_c_*(*t*_max_) (*c*_min1_ = 0.23). Scenario 1 was found to be optimal at an intermediate parameter space (*c*_min1_ = 0.26/0.62, *c*_min2_ = 0.52/0) for *I*_max_ and *I_c_*(*t*_max_), respectively. Increases in the duration of intervention 1 allowed greater reductions to *I*_max_ and *I_c_*(*t*_max_) for a given *c*_min1_/*c*_min2_ parameter space, relative to baseline parameters (electronic supplementary material, figures S11–S15). The exception was scenario 3, with an increased intervention 1 duration also increasing *I*_max_ and *I_c_*(*t*_max_) obtained over the explored *c*_min1_/*c*_min2_ parameter space.

## 4. Discussion

This study adds to the current epidemiological modelling work [9–14] to explore the concept of NPI optimization. We identified an optimal parameter space for all considered intervention scenarios, with each scenario capable of minimizing both *I*_max_ and *I_c_*(*t*_max_) for a given set of optimal parameter values. However, we note that the exact value of the optimal parameter space is highly nuanced, and often highly sensitive to changes to the explored model parameters.

The optimal parameter space was found to be strongly influenced by the balance between the intervention peak timing and *c*_min_. Matching the timing of an intervention to the epidemic peak has been explored previously [9,10]. However, we demonstrate that it is also necessary to match the timing of the epidemic peak with the greatest magnitude of the intervention (*c*_min_/*c*_min1_/*c*_min2_) if reductions to *β*(*t*) vary. This can be intuitively observed by comparing scenario 2 (*c*_min_ at *t*_p_) and scenario 3 (*c*_min_ at *t*_p_ + *d_t_*) (figure 2), with scenario 2 being optimal at a later trigger point to coincide with the early *c*_min_ reduction and scenario 3 optimal with an earlier trigger to coincide with the later *c*_min_ reduction. We also note the existence of optimal intermediate *c*_min_ values facilitating the build-up of infection-induced, protective immunity during the intervention. This phenomenon is well reported in modelling literature, with time-limited interventions found to be optimal when *R*_e_(*t*) is maintained near the threshold for sustained transmission (*R*_e_(*t*) ≈ 1) [25].

However, attaining these optima in practice is likely to be difficult [10]. The ongoing COVID-19 outbreak has highlighted the limited capacity of policymakers to effectively micromanage the course of an outbreak [29]. Factors such as varying public compliance, imperfect disease surveillance, policy miscommunication, confounding parallel interventions and implementation lag between the introduced interventions and observable changes in disease prevalence will contribute to large levels of intervention implementation error [8,30,31]. If placed in the context of the narrow parameter optima observed throughout this study, these effects will likely have substantial epidemiological consequences.

An alternative approach could involve interventions that are more robust to implementation error, but less efficacious than the theoretically optimal intervention. An example of this can be observed in the single-intervention sensitivity analysis, with longer-than-optimal interventions providing robust, but less efficacious reductions to *I*_max_ and *I_c_*(*t*_max_) for scenarios 3 and 4 relative to their optimal *t*_dur_ value (figure 1*b*). Owing to the relative insensitivity of these robust interventions to imperfect parameter choices, these intervention strategies will likely excel in uncertain real-time outbreaks where the current epidemiological situation is often unknown. Parallels of these interventions can be observed in the ongoing COVID-19 outbreak, with recurring themes of ‘hit it hard and fast’ providing simple, yet robust advice to policymakers [15,32].

We note that for a single time-limited intervention, it was not always optimal to intervene at a maximal strength or duration. For example, increasing *t*_dur_ for scenario 3 shifted the timing of *c*_min_ past the epidemic peak, lessening the impact of the NPI relative to optimal *t*_dur_ (figure 1). Additionally, NPIs were assumed to be time-limited, with even maximal- strength interventions ending before the termination of the model simulation, allowing rebounds in prevalence. This decision was made because of the infeasible nature of having an indefinite implementation of severe restrictions owing to societal disruptions. However, we note that when considered in the context of a robust, but suboptimal intervention, intervening maximally to increase *t*_dur_ and minimize *c*_min_ can be considered the most efficacious strategy to reduce *I*_max_ or *I_c_*(*t*_max_) (figures 1 and 2).

Similarly, for the multi-intervention scenario, an earlier and stronger intervention can provide reductions to *I*_max_ and *I_c_*(*t*_max_) under suboptimal circumstances as part of a robust intervention (figure 3). However, this only holds true in the context of the initial intervention, with this acting as a delaying action, allowing successive interventions to compensate and further reduce *I*_max_ and *I_c_*(*t*_max_). This highlights the additional role of robust interventions to permit future decision-making when the current epidemiological situation is uncertain. This has practical consequences, with policymakers able to use an earlier intervention to delay the epidemic peak if the optimal intervention strategy is unknown, providing time for the build-up of healthcare capacity and the opportunity for later interventions to course-correct.

We note that population ‘lockdown’ measures have been presented as an integral part of a package of measures, used to drive down the level of infection and ‘buy’ time for the introduction of more sustainable measures, such as contact tracing or vaccination [33,34]. We note that in the context of delaying the epidemic peak, it is universally optimal to introduce the initial ‘lockdown’ measures earlier, more strongly and for as long as necessary, until more sustainable intervention measures can be introduced indefinitely (electronic supplementary material, figure S16). However, this also highlights the importance of prioritizing the development of these sustainable measures, with the harsh consequences of severe, lengthy NPI measures making indefinite delaying actions expensive and ultimately unsustainable.

We note that an SIR model was one of many model structures considered to model the optimization of COVID-19 NPI strategies. An SEIR framework can be considered a more accurate description of the epidemiological characteristics of SARS-CoV-2, with a non-infectious ‘exposed’ state preceding infectiousness. We explored the inclusion of an exposed state, which shifted the timing of the optimal intervention to a later point, but did little to change the qualitative patterns observed when using the SIR framework (electronic supplementary material, figure S17). An assumption of lifelong immunity was also made following SARS-CoV-2 infection. We note that inclusion of a SIRS framework with differing levels of waning immunity would result in negligible impact on the optimal parameter space to minimize *I*_max_ relative to the original SIR model (electronic supplementary material, figure S18). This is likely due to the similar importance of controlling the initial epidemic wave to minimize *I*_max_ for both SIR and SIRS models. However, a different optimal parameter space to minimize *I_c_*(*t*_max_) was observed, likely as a result of the replenishment of susceptibles and long-term endemicity of COVID-19. Care must, therefore, be taken when interpreting the results of this study with regard to the long-term dynamics of COVID-19, especially owing to the ongoing uncertainty regarding the presence of long-term immunizing infection following SARS-CoV-2 infection. Owing to the large levels of uncertainty with regard to the immunological and transmission characteristics of SARS-CoV-2, and with the aim of this study to describe the existence and qualitative patterns of intervention optima, and not forecast or describe the exact timing, the use of a SIR model was deemed justifiable for this analysis. However, we note that for future predictive models that look to accurately identify the optimal parameter space, the integration of potentially all of the aforementioned model structures will likely be necessary using a SEIIRS or SEIRS model.

The socio-economic cost of each intervention was also not considered in this analysis. Factors such as adherence have a large impact on intervention efficacy [23], and the inclusion of these factors in the model may potentially provide support for NPI strategies with dedicated ramping or pulsing periods, which aim to partially mitigate the socio-economic effects and societal disruption of strong NPIs. A relatively simple disease metric was also used for this study, with an optimal intervention able to reduce the maximum peak prevalence, *I*_max_, and attack rate, *I_c_*(*t*_max_). While outside of the scope of this study, the use of other epidemiologically relevant outcome measures such as occupied ICU capacity or mortality per 100 000 population may be of interest when investigating optimal COVID-19 interventions in a more policy-relevant context. This could also be complemented by an exploration into the impact of individual or population-level variation of risk on intervention optimization [35–37].

NPI optimization has been highlighted in this study as a powerful tool to greatly mitigate the epidemiological impacts of a COVID-19 outbreak. This can be considered of significant relevance, with the recent reinstitution of NPIs and stricter measures being used to combat resurgent outbreaks. However, the results described in this study are highly nuanced, with narrow intervention optima and a number of other factors likely preventing the trajectory of an epidemic conforming uniformly to the dynamics observed in this study. We highlight robust interventions as an alternative policy option, with these interventions being less prone to implementation error but potentially suboptimal compared with theoretically optimal strategies. These interventions have the additional benefit of being a risk-averse approach, often favourable during the initial stages of the outbreak, where the impact of risky public health policy can lead to disastrous consequences. We also note that the sensitivity analysis-based framework used in this study is applicable to other immunizing pathogens, where transmission can be mitigated through NPIs. This approach has particular benefit in emerging outbreak scenarios where the rapid identification of optimal (or even robust) NPI implementation can contribute to the evidence base for outbreak response and reduce indecisiveness.

Finally, we stress that it was not the intention of this study to propose any one strategy as a singular policy option for COVID-19 control. The evidence from this study should be taken into context with the work tirelessly undertaken by the wider epidemiological and modelling community. It is only through this collaboration and synthesis that effective and altruistic public health policy can be generated to combat the COVID-19 pandemic.

## 5. In context

The work presented in this manuscript was originally submitted as a series of three technical briefings to SPI-M in early-March 2020. The study was motivated by the prevailing need to understand the potential impact resulting from severe non-pharmaceutical interventions on the transmission dynamics of COVID-19 and the consequences of releasing NPIs. We note that this work was conducted before the initiation of the first UK lockdown (23 March 2020). These three technical briefings were as follows:

1. Technical briefing 1—Time-limited social distancing measures and the shape of the epidemic curve (29 February 2020).
2. Technical briefing 2—Optimizing trigger times for social distancing measures (SDMs) (4 March 2020).
3. Technical briefing 3—Uncertainty about timings of SDMs (4 March 2020).

The original core analysis conducted for SPI-M utilizsed parameter values that were contemporary as of March 2020. This includes an *R*_0_ value that was later updated from 2 to 2.8 in the formalized manuscript in July/August 2020 [17]. This reflects the substantial improvements in knowledge regarding the epidemiology and immunological characteristics of COVID-19 in this later period.

## Data accessibility

Code used to generate/run all model simulations in this study can be found in the GitHub repository: https://github.com/alexmorgan1995/NPI_Analysis.
